# Using sequential bone cutting to titrate soft tissue balance in total knee arthroplasty effectively minimizes soft tissue release

**DOI:** 10.1186/s12891-023-07005-5

**Published:** 2023-11-08

**Authors:** Mingxue Chen, Dejin Yang, Hongyi Shao, Shouwei Rui, Yuefeng Cao, Yixin Zhou

**Affiliations:** Department of Orthopaedic Surgery, Beijing Jishuitan Hospital, Capital Medical University, No.31 Xinjiekou East Street, Xicheng District, Beijing, 100035 China

**Keywords:** Total knee arthroplasty, Sequential bone cutting, Soft tissue balance, Soft tissue release

## Abstract

**Background:**

Achieving soft tissue balance while maintaining limb alignment within acceptable boundaries is crucial for successful total knee arthroplasty (TKA). We proposed a sequential bone cutting (SBC) technique to titrate the soft tissue balance in robot-assisted TKA to achieve the desired balance with minimum soft tissue release.

**Methods:**

In total, 106 robot-assisted TKAs using the SBC technique were included. The preoperative hip-knee-ankle angle (HKA) was < 10° in 76 and ≥ 10° in 30 knees. The gaps were initially balanced with the help of the pre-resection balancing provided by the robotic system. Soft tissue balance and alignment were quantitatively measured after the initial bone cutting and final bone cutting. Additional adjustments (bone recuts and soft tissue releases) required to address soft tissue imbalance after initial bone cutting were recorded. The frequencies of soft tissue releases, soft tissue balance, and resultant alignment ≤ 3° were compared between non-severe (HKA < 10°) and severe deformity (HKA ≥ 10°) groups.

**Results:**

Soft tissue balance was achieved in 45 knees (42.5%) after initial bone cutting and in 93 knees (87.7%) after final balancing. The postoperative alignment was within 3° from neutral in 87 knees (82.1%) and 3–5° in 17 knees (16.0%). For unbalanced knees (n = 61) after initial bone cutting, soft tissue release was avoided by SBC in 37 knees (60.7%) and was deemed necessary in 24 knees (39.3%). Soft tissue release was more likely to be avoided in the non-severe deformity cohort (86.8% [33 of 38]) than in the severe deformity cohort (17.4% [4 of 23]; p < 0.001). The non-severe deformity cohort showed a significantly higher rate of resultant alignment ≤ 3° from neutral than the severe deformity cohort (90.8% vs. 60.0%; p < 0.001).

**Conclusion:**

Pre-resection balancing is inappropriate to ensure soft tissue balance. The SBC technique is effective in minimizing soft tissue release while maintaining overall alignment within acceptable boundaries.

## Introduction

Although instruments and implants have improved continuously, the dissatisfaction rate following total knee arthroplasty (TKA) remains as high as 15-20% [1, 2]. Malalignment and soft tissue imbalance are important causes of poor function and premature failure [3, 4]. Achieving soft tissue balance while maintaining lower limb alignment within acceptable boundaries is crucial for successful TKA [5].

Neutral mechanical alignment has been the gold standard for TKA for decades [6]. However, subsequent soft tissue releases are more likely required to balance the knee for target alignment after bone resection [7]. Although soft tissue release may help correct unbalanced gaps, it lacks precision and reproducibility and may have some unexpected side effects, such as excessive laxity, joint instability, abnormal kinematics, weakening of ligaments, and residual pain. [8]. Recent studies have indicated that minor mechanical axis undercorrection can minimize soft tissue release during TKA. Vanlommel et al. [9] found that a slight undercorrection in varus knees contributed to less ligament release, resulting in less pain and satisfactory long-term results. It is important to note that most joint surgeons perform bone cutting and soft tissue releases independently to balance the knee joint with conventional instruments. Although surgeons have recognized the interplay between alignment and soft tissue balance, the relationship is still decoupled during the surgical process [10].

Robotic technology helps surgeons perform bone cutting precisely and achieve alignment more accurately [11]. A robot also allows surgeons to initially balance gaps by altering the bone cutting angle slightly before making any bone cuts, which is known as pre-resection balancing [12, 13]. However, because the pre-resection soft tissue envelope status cannot be properly gauged, especially for severe deformities, pre-resection balancing cannot guarantee final balancing after a bone cut is executed [14].

To achieve optimal soft tissue balance while keeping lower limb alignment within acceptable boundaries, we proposed an original technique known as sequential bone cutting (SBC) in this article. The primary aim of this study was to determine whether pre-resection balancing was adequate to ensure final soft tissue balance. A secondary aim was to determine whether the SBC technique helped minimize soft tissue release while maintaining overall alignment with acceptable boundaries.

## Patients and methods

### Patient selection

This retrospective study was approved by the hospital’s ethics committee (approval No. K2023-008-00). Between May 2021 and November 2022, a total of 111 consecutive patients had robotic-assisted TKA (Mako, Stryker Corp, Mahwah, NJ, USA) using the SBC technique at our institution. All procedures were performed by the same experienced surgeon (YZ). A cruciate retaining (CR) knee implant system (Triathlon, Stryker Corp) was used in all cases.

The inclusion criteria included varus osteoarthritis of the knee undergoing primary robot-assisted TKA using the SBC technique. The exclusion criteria were: previous knee surgery (two knees) and lack of intraoperative measurement data (three knees). The remaining 106 knees (106 patients; 85 females and 21 males) were included in this study (Table 1). The patients had a mean age of 67.6 years (standard deviation (SD) 6.8), with a mean body mass index of 26.8 kg/m^2^ (SD 3.7). We measured the preoperative hip-knee-ankle axis angle (HKA) on full-length standing radiographs to assess the severity of deformities. Preoperative alignment was divided into two subgroups: non-severe deformity (HKA < 10°) and severe deformity (HKA ≥ 10°) [15]. The preoperative HKA was 7.6 ± 3.9° in varus. The preoperative HKA was < 10° in 76 (68.1%) knees and ≥ 10° in 30 (31.9%) knees. Patient demographics between the non-severe and severe deformity groups are shown in Table 1.

**Table 1:** Patient demographics

Variable	Total (n = 106)	Non-severe (n = 76)	Severe (n = 30)	p-value
Sex, female, n (%)	85 (80.2)	61 (80.3)	24 (80.0)	1.000
Side, left, n (%)	57 (53.8)	44 (57.9)	13 (43.3)	0.199
Mean age, yrs (range; SD)	67.6 (50 to 87; 6.8)	67.5 (53 to 82;6.4)	66.8 (50 to 87;7.8)	0.642
Mean BMI, kg/m^2^ (range; SD)	26.8 (19.2 to 41.5; 3.7)	26.7 (19.2 to 39.4;3.8)	27.1 (21.5 to 41.5;3.5)	0.647
Preoperative flexion contracture, ° (range; SD)	10.2 (-10 to 23; 5.6)	9.7 (-4 to 20; 5.0)	11.5 (-10 to 23;7.0)	0.131
Preoperative HKA, ° (range; SD)	7.6 (1.5 to 19; 3.9)	5.7 (1.5 to 9; 2.0)	12.6 (10 to 19; 2.7)	< 0.001

BMI, body mass index; HKA, hip-knee-ankle angle

### Surgical technique

**Fig. 1:**
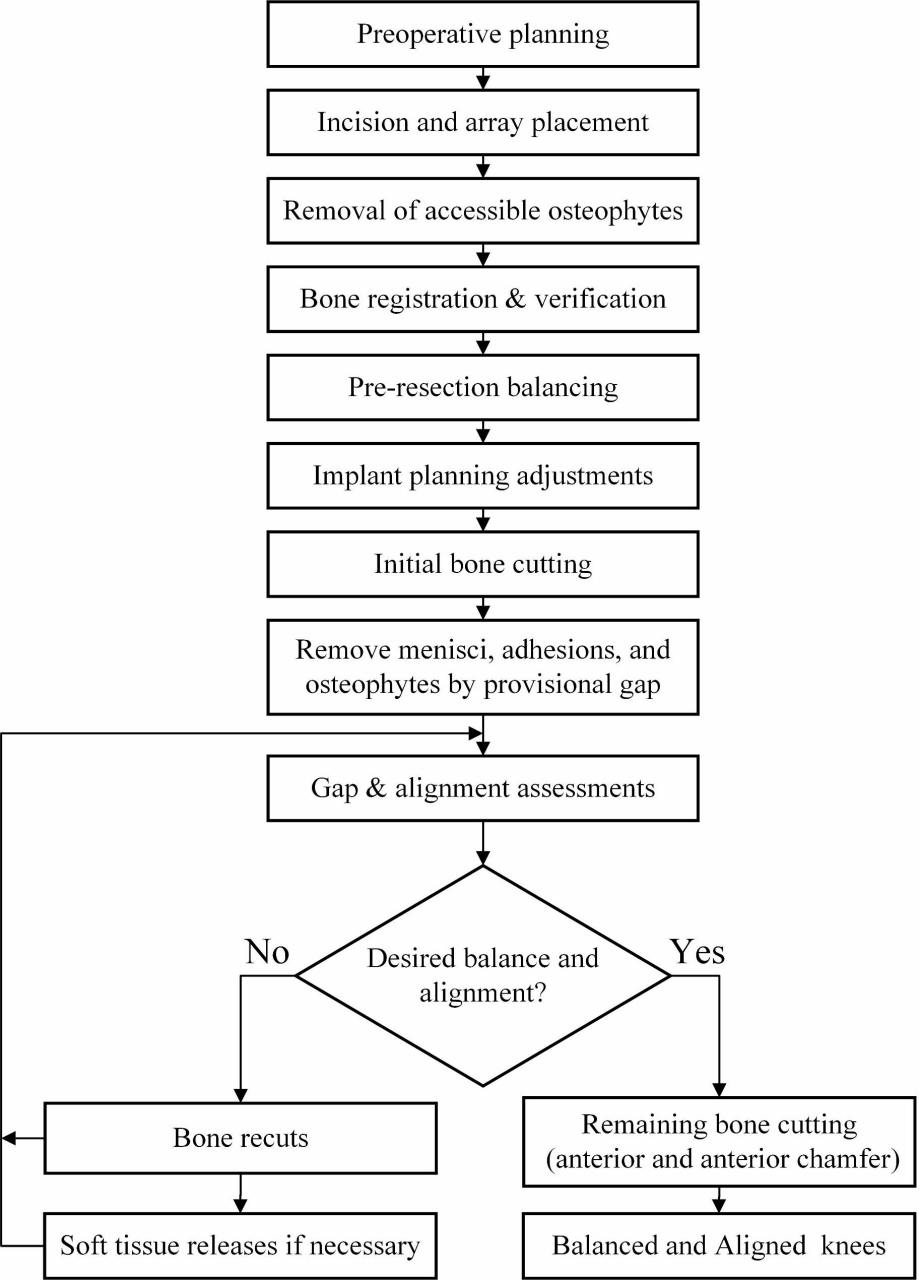
SBC technique workflow for soft tissue balancing

The workflow of the SBC technique is illustrated in Fig. 1. A standard anterior midline longitudinal skin incision with a medial parapatellar arthrotomy was used to expose the knee joints. Accessible osteophytes were removed routinely at this stage. After bone registration, we performed a varus/valgus stress test in extension and inserted a spoon at 90° of flexion to assess soft tissue laxity. Based on the pre-resection assessment, we adjusted the component position to balance extension and flexion gaps and maintain lower limb alignment within a 3° deviation of the neutral mechanical alignment. Pre-resection balancing allows surgeons to balance the planned gaps before bone cutting. Our goal was to create a 16-mm rectangular gap in flexion and extension at this stage.

**Fig. 2:**
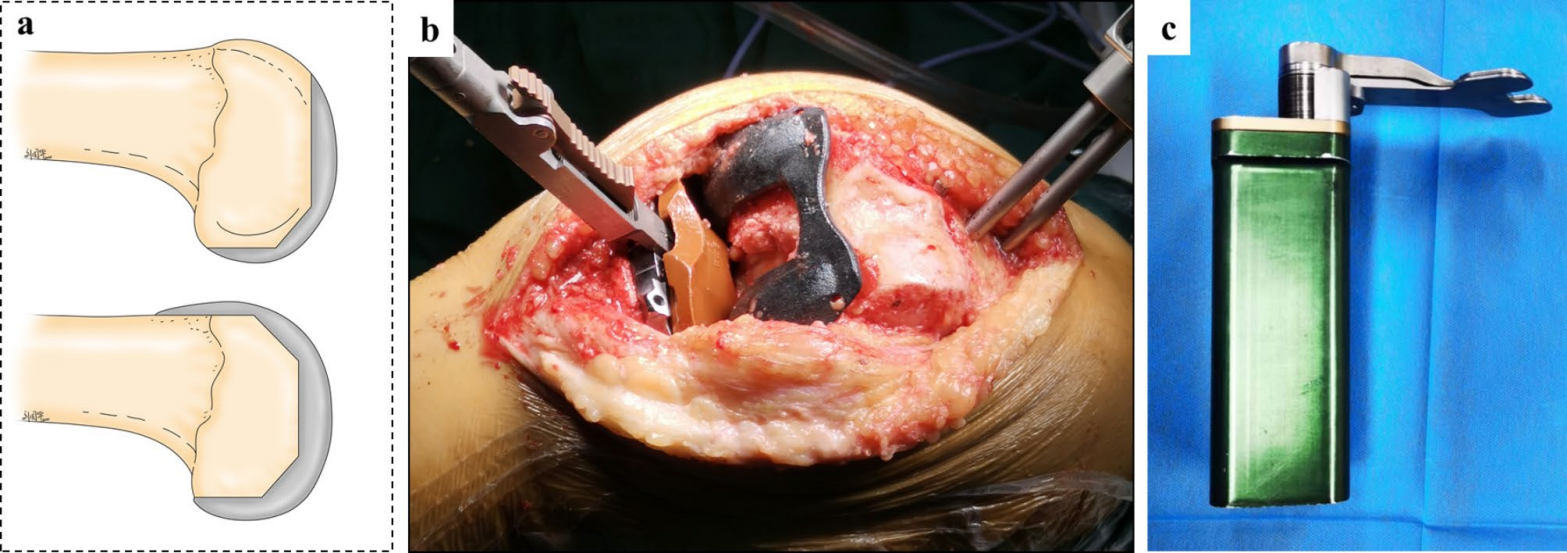
**(a)** Initial-cutting trial and femoral component, **(b)** an initial-cutting trial placement on the femur during operation, **(c)** a digital tensor (Tinavi Corp., Beijing, China)

With the SBC technique, initial bone cutting (distal femur, posterior chamfer femur, posterior femur, and tibia) was performed with robotic arm assistance, and provisional extension and flexion gaps were created. Through a provisional gap, we removed the meniscal remnants and posterior osteophytes. Femoral initial-cutting trials were designed to account for the various sizes of the femur and fit the SBC technique (Fig. 2a,b). It has the same surface geometry as the Triathlon femoral component but with no anterior flange and anterior chamfer parts. The thickness of the initial-cutting trial was 2 mm thinner than the Triathlon femoral component. After setting the initial-cutting trial on the femur, a digital tensor (Tinavi Corp., Beijing, China) (Fig. 2c) was inserted into the knee joint to tension the gaps, with the patella reduced. Gaps were measured with tension forces at 10° (extension) and 90° (flexion) to quantitatively assess the state of soft tissue balance. The overall limb alignment was checked using the robotic “real-time” values.

**Fig. 3:**
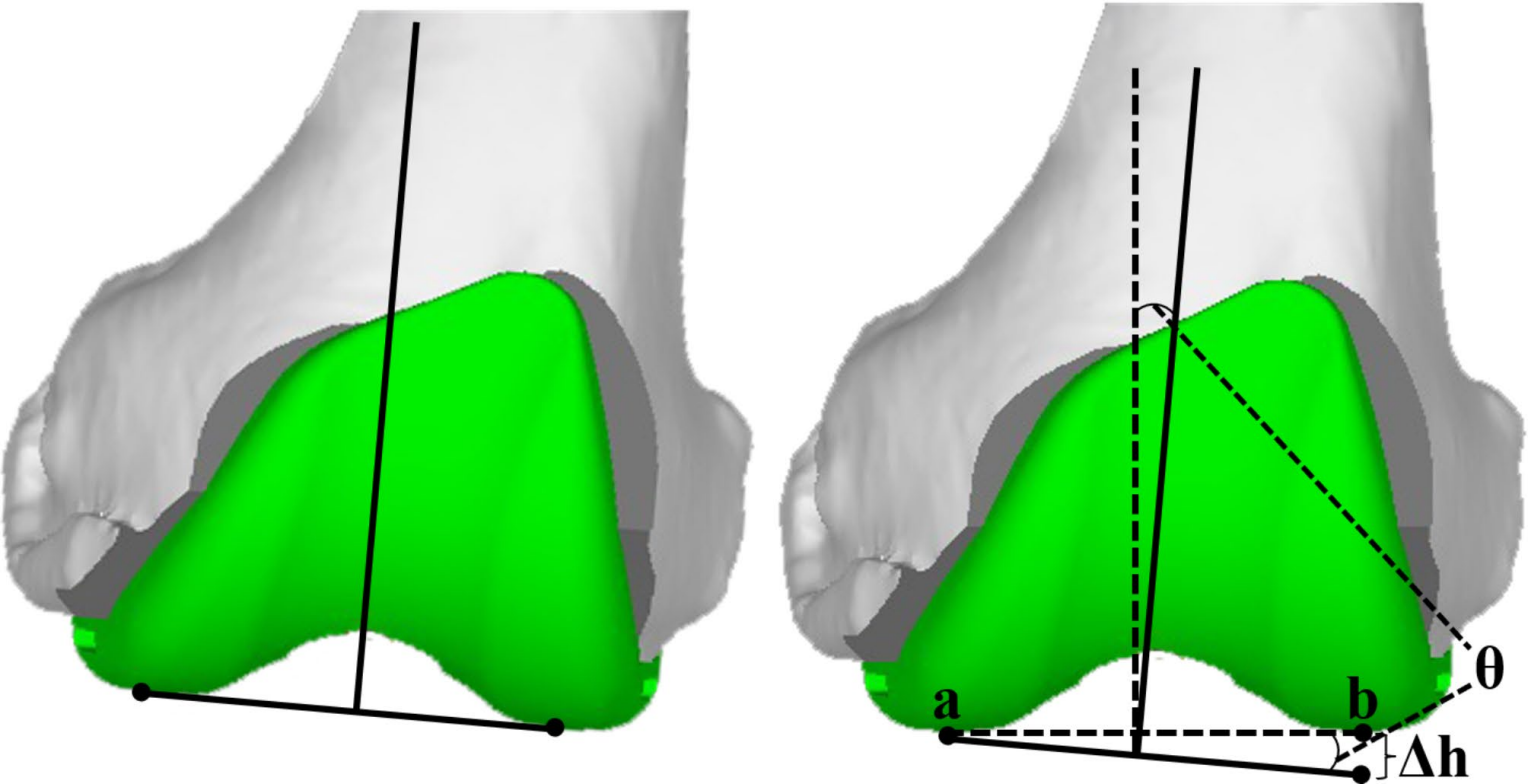
Schematic diagram of the relationship between gap and alignment by angular bone recuts

We defined a balanced knee as mediolateral gap differences in flexion ≤ 2 mm, mediolateral gap differences in extension ≤ 1 mm, and flexion-extension gap differences ≤ 2 mm. We accepted limb alignment within 3° of the neutral mechanical alignment. For severe varus deformities, we tolerated an overall limb alignment limit of 5° and used a tibial stem extension. If alignment and gaps were acceptable, we performed an additional 2-mm-thick distal and posterior femur (compensation for the under-resection during initial bone cutting) and the remaining bone resections (anterior femur, anterior and posterior chamfer femur). If the mediolateral gaps were unbalanced, angular bone recuts (recuts in varus/valgus or external/internal rotation) were performed with robotic system assistance to symmetrize the gaps, as long as the limb alignment was within the acceptable alignment boundaries (Table 2). The angle was calculated using the following balance equation: Δh = Sinθ·W_ab_, where W_ab_ is mediolateral width (Fig. 3). When 0°≤ n ≤ 5°, it obeyed the following equation: sin n = n sin1°. Generally, a 1° change in the coronal alignment of the distal femur resulted in a 1-mm difference in the gap of one side. For the extension-flexion gap imbalance, parallel bone recuts were made to equalize the extension and flexion gaps. Soft tissue release is considered necessary only if a balance cannot be achieved by bone recuts within the alignment boundaries based on the judgment of the operating surgeon. The soft tissue balance was rechecked with femoral trial implants. Any bone recuts and soft tissue releases were recorded. In this study, we defined soft tissue releases as any impact on the soft tissue after initial bone cutting.

**Table 2:** Bone recut algorithm

	In flexion	In extension	Both in flexion and extension
Tight medial gap	Posterior femur recut in external rotation	Distal femur recut in varus	Tibia recut in varus
Tight lateral gap	Posterior femur recut in internal rotation	Distal femur recut in valgus	Tibia recut in valgus
Tight medial and lateral gaps	Tibia recut with greater slope and/or posterior femur parallel recut	Distal femur parallel recut	Tibia parallel recut

### Statistical analysis

All statistical analyses were conducted using SPSS (version 22.0; IBM Corporation, Armonk, New York, USA). Continuous variables between the two groups were compared using an independent-sample t-test (data were normally distributed) or the Mann–Whitney U test (data were abnormally distributed). Categorical variables were analyzed using Pearson’s chi-square test or Fisher’s exact test. Statistical significance was set at p < 0.05.

## Results

Of the 106 knees, the gap differences after initial bone cutting and final balancing are shown in Table 3. After initial bone cutting, mediolateral gap differences in extension were within 1 mm in 67 knees (63.2%), 1–2 mm in 20 knees (18.9%), and > 2 mm in 19 knees (17.9%). In flexion, the mediolateral balance was achieved within 1 mm in 56 knees (52.8%), 1–2 mm in 23 knees (21.7%), and > 2 mm in 27 knees (25.5%). Gap difference in flexion–extension within 1 mm was observed in 61 knees (57.5%), 1–2 mm in 21 knees (19.8%), and > 2 mm in 24 knees (22.7%). According to the criteria described, soft tissue balance after initial bone cutting was achieved in 45 knees (42.5%). The balance rate was significantly higher in the non-severe deformity group (50.0% [38 of 76]) than in the severe deformity group (23.3% [7 of 30]; p = 0.016).

Eventually, mediolateral gap difference in extension was achieved within 1 mm in 98 knees (92.5%) and 1–2 mm in 8 knees (7.5%). Mediolateral gap differences in flexion within 1 mm were observed in 99 knees (93.4%) and 1–2 mm in 7 knees (6.6%). For flexion-extension gap difference, 81 knees (76.4%) were within 1 mm, 21 knees (19.8%) were 1–2 mm, and 4 knees (3.8%) were > 2 mm. A total of 93 knees (87.7%) met the criteria for soft tissue balance. The overall balance rate was significantly higher in the non-severe deformity group (92.1% [70 of 76]) than in the severe deformity group (76.7% [23 of 30] p = 0.046). The postoperative alignment was within 3° in 87 knees (82.1%) and 3–5° in 17 knees (16.0%). Of these, the postoperative alignment of ≤ 3°, 3–5° and > 5° was achieved in 90.8%, 9.2%, and 0% of cases for the non-severe deformity cohort and 60.0%, 33.3%, and 6.7% of cases for severe deformity cohort, respectively, which was statistically significant (p < 0.001).

**Table 3:** Gap differences after initial bone cutting and final balancing

Variable	Initial bone cutting							Final balancing			
	Total	Non-severe (n = 76)	Severe (n = 30)	p-value			Total		Non-severe (n = 76)	Severe (n = 30)	p-value
M-L gap differences in extension, n (%)				0.004							0.685
≤ 1 mm	67 (63.2)	54 (71.1)	13 (43.3)				98 (92.5)		71 (93.4)	27 (90.0)	
1–2 mm	20 (18.9)	14 (18.4)	6 (20.0)				8 (7.5)		5 (6.6)	3 (10.0)	
> 2 mm	19 (17.9)	8 (10.5)	11 (36.7)				0 (0)		0 (0)	0 (0)	
M-L gap differences in flexion, n (%)				0.686							0.401
≤ 1 mm	56 (52.8)	42 (53.3)	14 (46.6)				99 (93.4)		72 (94.7)	27 (90.0)	
1–2 mm	23 (21.7)	15 (19.7)	8 (26.7)				7 (6.6)		4 (5.3)	3 (10.0)	
> 2 mm	27 (25.5)	19 (25.0)	8 (26.7)				0 (0)		0 (0)	0 (0)	
F-E gap differences, n (%)				0.194							0.035
≤ 1 mm	61 (57.5)	45 (59.2)	16 (53.3)				81 (76.4)		63 (82.9)	18 (60.0)	
1–2 mm	21 (19.8)	17 (22.4)	4 (13.3)				21 (19.8)		12 (15.8)	9 (30.0)	
> 2 mm	24 (22.7)	14 (18.4)	10 (33.4)				4 (3.8)		1 (1.3)	3 (10.0)	
Overall balance, n (%)	45 (42.5)	38 (50.0)	7 (23.3)	0.016			93 (87.7)		70 (92.1)	23 (76.7)	0.046

M-L, medial-lateral; F-E, flexion–extension

For unbalanced knees (n = 61) after initial bone cutting, soft tissue release was avoided by SBC in 37 knees (60.7%) and was deemed necessary in 24 knees (39.3%). Of the 24 knees that required soft tissue releases, 5 knees (13.2%) and 19 knees (82.4%) were in non-severe and severe deformity groups, respectively. Soft tissue release was more likely to be avoided in the non-severe deformity cohort (86.8% [33 of 38]) than in the severe deformity cohort (17.4% [4 of 23]; p < 0.001) (Table 4). Of the 61 unbalanced knees, 46 knees (75.4%) underwent one bone recut, 14 knees (23.0%) underwent two bone recuts, and 1 knee (1.6%) underwent three bone recuts. Patients with severe deformities required more bone recuts than those with non-severe deformities (1.5 vs. 1.1; p = 0.002) (Table 4). Thirty-four knees (55.7%) required a femoral recut only, and 27 knees (44.3%) required both tibial and femoral recuts. More specifically, over 90% (40 of 44) of distal femur recuts were performed within 2 mm (1.6 ± 0.5 mm, 0.5–2.5 mm), approximately 75% (35 of 47) of posterior femur recuts were performed over 2 mm (2.4 ± 0.8 mm, 1.0–5.0 mm), and all tibia recuts were performed within 2 mm (1.0 ± 0.5 mm, 0.5-2.0 mm) (Table 5). All angular bone recuts were performed within an angle of 2°. The femoral component was downsized in 15 cases. The average final tibial insert thickness was 9.5 ± 1.0 mm. The tibial insert for 9 mm was used in 80 knees (75.5%), 11 mm in 23 knees (21.7%), and 13 mm in 3 knees (2.8%). The mean surgical time from skin incision to wound closure was 102 ± 21 min.

**Table 4:** Additional adjustments of the unbalanced knees between non-severe and severe deformity groups

Variable	Total (n = 61)	Non-severe (n = 38)	Severe (n = 23)	p-value
Soft tissue releases, n (%)	24 (39.3)	5 (13.2)	19 (82.4)	< 0.001
Bone recuts, n (%)				0.002
1	46 (75.4)	34 (89.5)	12 (52.6)	
2	14 (23.0)	4 (10.5)	10 (42.1)	
3	1 (1.6)	0 (0)	1 (5.3)	

**Table 5:** The thickness of bone recuts

	0 mm	0.5 mm	1 mm	1.5 mm	2 mm	2.5 mm	3 mm	> 3 mm
Distal femur parallel recuts, n (%)	17 (27.9)	7 (11.5)	13 (21.3)	10 (16.4)	12 (19.7)	2 (3.3)	0 (0)	0 (0)
Posterior femur parallel recuts, n (%)	14 (23.0)	0 (0)	2 (3.3)	3 (4.9)	7 (11.5)	17(27.9)	16 (26.2)	2 (3.3)
Tibia parallel recuts, n (%)	45 (73.8)	2 (3.3)	9 (14.7)	2 (3.3)	3 (4.9)	0 (0)	0 (0)	0 (0)

## Discussion

Achieving soft tissue balance while maintaining lower limb alignment within acceptable boundaries is crucial for successful TKA. To achieve this goal, we proposed the SBC technique to address soft tissue imbalance. We found that even with pre-resection balancing, only 42.5% of knees achieved soft tissue balance after initial bone cutting. This was consistent with the findings of Bardou-Jaquet et al. [16] who reported that only 41% of cases were well-balanced after the first bone cut, based on the load sensor (Orthosensor). Gordon et al. [14] also reported that pre-resection planning targeting equal mediolateral gaps with a robotic system does not ensure balanced gaps after bone resection. It is more difficult to accurately measure soft tissue envelope status and achieve balancing before any bone cutting when cartilage, osteophytes, medial and lateral menisci, and adhesions are present. That is why pre-resection balancing is inappropriate to ensure soft tissue balance. Hence, additional adjustments (bone recuts or soft tissue releases) are often necessary after the initial bone cutting.

With the SBC technique, soft tissue release was avoided in 60.7% of cases. The strength of a robot is its ability to perform reliable and reproducible bone recuts with great convenience, which is difficult, if not impossible, in conventional TKA. If the mediolateral gaps were unbalanced, it is easy to perform angular bone cutting, rather than soft tissue release, to symmetrize the gaps with robotic system assistance. Similar to differential calculus, SBC, which is facilitated by a robotic system, allows segmentizing and reorganizing of the TKA surgical process to achieve perfect soft tissue balance. There are still 24 knees (39.3%) required soft tissue releases in our study. Of these, 19 knees were in the severe deformity cohort (n = 23, 82.4%) and 5 knees were in the non-severe deformity cohort (n = 38, 13.2%). Soft tissue release was more likely to be required for severe deformities. This could be explained by a strong relationship between the requirement for soft tissue release and preoperative coronal alignment [14]. Another strength of the robot is that it provides real-time information on limb alignment. Knowledge of the alignment gives surgeons the option to make fine bone recut adjustments within acceptable ranges [12]. A 3°-5° window of mechanical axis deviation may help avoid or minimize soft tissue release during TKA. However, for knees with severe deformities, changing the component position within acceptable alignment boundaries may be insufficient for achieving balance. To avoid significant malalignment, these cases usually require additional soft tissue release. Thus, soft tissue release was more commonly performed in knees with severe deformities.

The soft tissue balance was significantly improved by the SBC technique. In this study, 87.7% of all TKAs met the defined balance targets. The postoperative alignment was within 3° in 87 knees (82.1%) and 3–5° in 17 knees (16.0%). The results demonstrated the effectiveness of the SBC technique in achieving the desired balance while maintaining alignment within acceptable ranges. Soft tissue balance was achieved by initial bone cutting and further bone recutting (SBC). During the initial bone cutting, the distal and posterior femurs were under-resected by 2 mm. A provisional gap could provide surgeons with access to remove meniscal remnants, posterior osteophytes, and adhesions, which mainly influence the gaps in extension. After that, the provisional gap allowed for accurate gap measurement and soft tissue assessment under tension forces. These data could assist surgeons in decision-making and guide further bone recutting to reverse the tendency of gap imbalance or fine-tune gap balance. The SBC technique may lead to small increases in minutes of surgical time when compared to standardized robotic technique. The slight increase in surgery time was mainly taken for soft tissue assessment with the digital tensor and bone recutting with robotic arm assistance, which allowed surgeons to achieve soft tissue balancing with minimum soft tissue injury and more bone conserving. The resultant alignment was 3°-5° in 17 knees (16%) in this series. This may be attributed to the surgeon’s preference for bone recutting to address the imbalance at the cost of alignment. A previous study also reported that residual varus alignment did not compromise the results of TKAs in patients with preoperative varus [17].

After initial bone cutting, the average thickness of bone recuts was 1.6 mm on the distal and 2.4 mm on the posterior parts of the femur, respectively. It indicated that the extension gap and flexion gap might mismatch guided by the pre-resection balancing technique in robot-assisted TKAs with CR implants. That may explain why orthopedic surgeons feel that the flexion gap is tighter than the extension gap in robot-assisted TKAs, which differs from conventional TKAs. But this trend probably won’t be the same in robot-assisted TKAs with posterior-stabilized implants. All angular bone resections were performed within an angle of 2°. Moore et al. [18] also reported that the knee can move from an imbalanced state to a balanced state with adjustments within 2° or 2 mm. In general, these findings were in accordance with the balance equation mentioned above. Therefore, a 2 mm under-resection could contribute to leaving enough room for further bone recut adjustment and avoiding over-resection.

It should be noted that while the SBC technique contributes to achieving soft tissue balance defined by us, a generally accepted definition of a balanced knee arthroplasty is still absent [19]. To date, the notion that flexion and extension gaps are equal and symmetrical remains the primary goal in TKA [20]. However, it is still unclear whether a balanced knee is the best goal for a perfect TKA [21]. Because, a native knee is intrinsically lax on the lateral side relative to the medial side (especially in flexion), which differs from the proposed balanced knees [22]. The kinematic alignment technique respects this soft tissue laxity and aims to restore the anatomy of the native knee [23]. However, solid evidence is currently lacking to confirm the clinical effectiveness of this alignment technique. Hence, more long-term clinical data with larger numbers are warranted to illustrate the relationship between the balancing target and clinical outcomes, and further guide how to achieve the target.

Our study had several limitations. First, this was a retrospective study with inherent weaknesses, including recall and selection biases. Second, this study presented the results of a single-surgeon case series. Personal preferences, specific techniques, and surgical strategies may have confounded our results. Third, functional outcomes were not included in this study, although follow-up is ongoing, and the relationship between the desired balancing target and functional outcomes will be considered for future publications.

## Conclusion

The pre-resection balancing was inappropriate to ensure soft tissue balance. Additional adjustments (bone recuts or soft tissue releases) are often necessary after the initial bone cutting. The SBC technique is effective in minimizing soft tissue release while keeping alignment in range. For knees with significant deformities, soft tissue releases, and more bone recuts are likely to be required to balance the knees. The non-severe deformity group showed higher rates of final soft tissue balance and resultant alignment ≤ 3° compared with the severe deformity group.

## Data Availability

Data are available from the first author (Mingxue Chen, chenmingxueplagh@hotmail.com) on reasonable request and with permission of the hospital’s ethics institutional review board.
